# Paediatric inflammatory multisystem syndrome temporally associated with SARS-CoV-2 (PIMS-TS): a Brazilian cohort

**DOI:** 10.1186/s42358-022-00237-4

**Published:** 2022-02-21

**Authors:** André Cavalcanti, Aline Islabão, Cristina Magalhães, Sarah Veloso, Marlon Lopes, Rogério do Prado, Bruna Aquilante, Ana Maria Terrazas, Maria Fernanda Rezende, Gleice Clemente, Maria Teresa Terreri

**Affiliations:** 1 Pediatric Rheumatology Unit, Hospital das Clínicas da Universidade Federal de Pernambuco, Av. Prof. Moraes Rego, 1235 - Cidade Universitária, Recife, PE CEP 50670-901 Brazil; 2 Pediatric Rheumatology Unit, Hospital da Criança de Brasília José Alencar, Brasília, DF Brazil; 3 Centro Universitário de Brasília, Brasília, DF Brazil; 4 Centro Universitário Faculdade de Medicina do ABC, Santo André, SP Brazil; 5 Hospital Estadual de Bauru, Bauru, SP Brazil; 6 Pediatric Rheumatology Unit, Universidade Federal de São Paulo, São Paulo, SP Brazil

**Keywords:** SARS-CoV-2, COVID-19, Paediatric inflammatory multisystem syndrome, Kawasaki disease

## Abstract

**Background:**

Paediatric inflammatory multisystem syndrome (PIMS) associated with severe acute respiratory syndrome coronavirus 2 (SARS-CoV-2) has been described since mid-April 2020 with the first reports coming from Europe. Our objective was to describe the characteristics of patients among the Brazilian population.

**Methods:**

A multicenter retrospective study was conducted with the participation of five pediatric rheumatology centers in Brazil during the period from March to November 2020. Children and adolescents with PIMS temporally associated with SARS-CoV-2 (TS) who met the definition criteria for the disease according to the Royal College of Paediatrics and Child Health were included. Demographic, clinical, laboratory, therapeutic characteristics and molecular and serological diagnosis of SARS-CoV-2 infection were described.

**Results:**

Fifty-seven children and adolescents with PIMS-TS were evaluated, 54% female, with a median age of 8 (3–11) years. Most (86%) were previously healthy, with asthma being the main comorbidity, present in 10% of the patients. Fever was the main manifestation, present in all patients, followed by mucocutaneous and gastrointestinal features, present in 89% and 81% of the patients, respectively. Myocarditis occurred in 21% of the patients and in 68% of them required intensive care. The Kawasaki disease phenotype occurred in most patients (77%). All patients had elevated inflammatory markers, with elevated CRP being the most found (98%). Anemia and lymphopenia were present in 79% and 72%, respectively. Laboratory evidence of SARS-CoV-2 was found in 77% of the patients, with 39% positive RT-PCR and 84% positive serology for SARS-CoV-2. An immunomodulatory treatment was performed in 91% of the patients, with 67% receiving intravenous immunoglobulin (IVIG) associated with glucocorticoid, 21% receiving IVIG, and 3.5% receiving glucocorticoid. The median length of hospitalization was 10 days.

**Conclusions:**

This study showed a high morbidity of PIMS-TS in Brazilian children, with a prolonged length of hospitalization and a high rate of admission to pediatric intensive care unit. Multicenter prospective studies are needed to assess the morbidity of the disease in the medium and long term.

## Introduction

At the beginning of the pandemic of coronavirus disease 2019 (COVID-19) in China, it was demonstrated that children and adolescents were less affected by the disease, showing a higher number of mild and asymptomatic cases, lower rates of hospitalization and deaths when compared with adults [1–3]. However, a few weeks after the arrival of the severe acute respiratory syndrome coronavirus 2 (SARS-CoV-2) in Europe and in the USA, there was a change in the clinical and epidemiological pattern of the infection in pediatrics. Case reports and case series described a new hyperinflammatory syndrome in previously healthy children and adolescents, requiring hospitalization, most of them in pediatric intensive care units [4–6]. These patients presented fever and involvement of several organs and systems, such as gastrointestinal, cardiovascular, mucocutaneous, and neurological symptoms, among others. Laboratory tests revealed intense inflammatory activity and coagulopathy [7, 8].

In order to alert the medical community to the early recognition of this new hyperinflammatory syndrome, government agencies and medical entities have developed definition criteria for the syndrome with different nomenclatures [9–12].

Interestingly, some of these patients presented clinical manifestations similar to Kawasaki disease (KD) [13, 14], KD shock syndrome with a significant myocardial involvement and macrophage activation syndrome, 2–4 weeks after the presumed SARS-CoV-2 infection. In other words, a new post-infectious syndrome with a broad phenotypic spectrum and strong epidemiological evidence of association with SARS-CoV-2 has been established in pediatric patients [5, 6, 15].

Because several case series in different countries have described an association between KD and PIMS-TS, we conducted this study to describe the main demographic, clinical, laboratory, and therapeutic characteristics of Brazilian children and adolescents with PIMS-TS, whether or not they presented a KD phenotype.

## Methods

### Patients and design

A retrospective, convenience sample cohort study was conducted including 5 pediatric rheumatology centers in Brazil during the period from March 2020 to November 2020. The inclusion criterion was the presence of the hyperinflammatory syndrome associated with SARS-CoV-2 according to the Royal College of Paediatrics and Child Health (RCPCH) criteria and a maximum age of up to 18 years and 11 months [9].

### Data collection

An electronic clinical chart was developed and standardized for data collection at the participating centers. Each patient's data was collected during hospital admission, maintaining anonymity. Demographic and clinical data included current age, gender, presence of comorbidities, and length of hospital stay. The following clinical manifestations were assessed: fever, mucocutaneous, gastrointestinal, respiratory, neurological, cardiovascular manifestations, presence of KD (complete or incomplete), and KD shock syndrome.

Updated 2017 American Heart Association criteria were used to define the complete or incomplete KD [16]. The KD shock syndrome was defined by a systolic arterial hypotension, 20% reduction in the initial systolic blood pressure or clinical signs of hypoperfusion [17]. The following laboratory changes were recorded during hospitalization (up to 48 h after the admission): blood count, acute phase reactants (C-reactive protein—CRP and erythrocyte sedimentation rate—ESR), ferritin and D-dimer, aspartate aminotransferase, alanine aminotransferase, lactate dehydrogenase (LDH) and troponin.

Molecular diagnosis of active SARS-CoV-2 infection was performed by RT-PCR technique in nasopharyngeal swab and serological diagnosis (IgG or IgM) by ELISA or immunochromatography technique. Reference values were evaluated according to local providers in each participating center. The following imaging examinations were evaluated: echocardiogram, chest X-ray or CT scan and abdominal ultrasound or CT scan. A decreased left ventricular ejection fraction (< 55%) and/or an increased troponin concentration were used as indirect signs of myocarditis [18].

The main treatments used were glucocorticoid, intravenous immunoglobulin (IVIG), vasoactive drugs, need for admission to the intensive care unit, and need for mechanical ventilation.

The statistical analysis was descriptive for the continuous variables, being expressed as means and standard deviation (SD) or medians and interquartile range (IQR), and for the categorical variables expressed in absolute and relative values. Comparisons between groups were made using the Fisher’s exact test for categorical variables and the Mann–Whitney *U* test for continuous variables. A *p* value of < 0.05 was chosen as cutoff for significance. Data were analyzed with SPSS (version 20.0).

## Results

Fifty-seven children and adolescents with PIMS-TS were included. The median age was 8 (3–11) years and there was a slight female predominance. Only 8 (14%) patients had comorbidities (Table 1).

**Table 1:** Clinical and demographic features of patients with paediatric inflammatory multisystem syndrome temporally associated with SARS-CoV-2

Characteristics	Patients (n = 57)
Age [median in years, (IQR)]	8 (3–11)
Age group—n (%)	
0–5 years	19 (33)
6–12 years	34 (60)
13–18 years	4 (7)
Female sex—n (%)	31 (54)
Comorbidities—n (%)	
Previously healthy	49 (86)
Asthma	6 (10)
Obesity	1 (2)
Juvenile idiopathic arthritis	1 (2)
Main symptoms—n (%)	
Fever	57 (100)
Mucocutaneous—n (%)	51 (89)
Rash	46 (80)
Conjunctivitis	42 (73)
Oral mucosal changes	36 (63)
Gastrointestinal symptoms	46 (81)
Abdominal pain	34 (60)
Vomiting	34 (60)
Diarrhea	23 (40)
Respiratory symptoms n (%)	21 (37)
Neurologic (headache) n (%)	14 (24)
Serositis n (%)	13 (23)
Pleuritis	5 (9)
Pericarditis	7 (12)
Peritonitis	6 (10)
Myocarditis n (%)	12 (21)
Organ-system involvement—n (%)	38 (67)
Gastrointestinal + mucocutaneous	42 (74)
Mucocutaneous + cardiovascular	27 (47)
Gastrointestinal + cardiovascular	25 (44)
Kawasaki disease n (%)	
Complete phenotype	32 (56)
Incomplete phenotype	12 (21)

*IQR* interquartile range

All patients had persistent fever prior to hospital admission. Gastrointestinal and respiratory symptoms occurred in 81% and 37% of the patients, respectively. Thirteen (23%) of the 57 patients had some form of serositis (pleuritis, pericarditis or peritonitis). Myocarditis occurred in 21% of the patients. Most of the patients (67%) had a progressive involvement of at least 3 organs or systems.

Regarding the phenotype of KD, 32 (56%) patients met the criteria for classic KD and 12 (21%) for the incomplete form of KD (Table 1).

Laboratory changes are summarized in Table 2. All patients revealed an increase of CRP with the exception of one, who showed an increase of ESR and D-dimer. Most revealed an increase of ESR, ferritin, aminotransferases, and LDH. An alteration of troponin and D-dimer was found in 16% and 96% of the patients, respectively. Anemia and lymphopenia were observed in more than 70% of the patients, and leukocytosis and thrombocytopenia were observed in more than 30% of the patients. Of the 49 patients who had RT-PCR testing, 39% were positive; and of the 45 patients who had serology testing, 84% were IgG and/or IgM positive. Alteration of RT-PCR and/or serology (IgG and/or IgM) occurred in 44/57 (77%) of the patients (Table 2).

**Table 2:** Laboratory tests and treatment of patients with paediatric inflammatory multisystem syndrome temporally associated with SARS-CoV-2

Laboratory test	Patients (n = 57)
Increased C-reactive protein—n/total n (%)	56/57 (98)
ESR ≥ 40 mm/h—n/total n (%)	47/50 (94)
Ferritin > 500 ng/ml—n/total n (%)	26/44 (59)
Aspartate aminotransferase ≥ 40 U/L—n/total n (%)	37/53 (70)
Alanine aminotransferase ≥ 40 U/L—n/total n (%)	36/53 (68)
Lactate dehydrogenase ≥ 300 U/L—n/total n (%)	24/44 (55)
Increased troponin n/total n (%)	8/49 (16)
Increased D-dimer n/total n (%)	45/47 (96)
Anemia (Hb < 11 g/dl)—n/total n (%)	45/57 (79)
Hemoglobin g/dl [median, (IQR)]	9.9 (8.3–10.8)
Leukocytosis (> 15.000 cells/mm^3^)—n/total n (%)	20/57 (35)
Leukopenia (< 4.000 cells/mm^3^)—n/total n (%)	7/57 (12)
Lymphopenia (< 1.500 cells/mm^3^)—n/total n (%)	41/57 (72)
Leukocytes/mm^3^ [median, (IQR)]	11.100 (7.480–21.100)
Neutrophils/mm^3^ [median, (IQR)]	8.300 (3.395–15.165)
Lymphocytes/mm^3^ [median, (IQR)]	1.001 (683–1.609)
Thrombocytosis (> 450.000 cells/mm^3^)—n/total n (%)	7/57 (12)
Thrombocytopenia (< 100.000 cells/mm^3^)—n/total n (%)	17/57 (30)
Platelets/mm^3^ [median, (IQR)]	126.000 (89.000–217.000)
RT-PCR (nasopharyngeal swab)—n/total n (%)	19/49 (39)
Serology	
IgG positive	32/45 (71)
IgM positive	1/45 (2)
IgG and IgM positive	5/45 (11)
RT-PCR positive and/or serology positive—n/total n (%)	44/57 (77)
Treatment	
Intravenous immunoglobulin + glucocorticoid n (%)	38/57 (67)
Intravenous immunoglobulin n (%)	12/57 (21)
Glucocorticoid n (%)	2/57 (3.5)

*ESR* erythrocyte sedimentation rate, *Hb* hemoglobin, *IQR* interquartile range, *RT-PCR* reverse transcription polymerase chain reaction

The main changes found in the echocardiogram were a reduction of the ejection fraction below 55% in 9 (16%) of the patients, an involvement of the right coronary artery in 4, of the left coronary artery in 2, and of the right and left coronary arteries in one patient. A pericardial effusion was found in 7 (12%) of the patients.

The main pulmonary changes in chest X-ray and/or CT scan were ground-glass opacity in 9 patients, a pleural effusion in 5, and atelectasis in 4. The main abdominal changes found in ultrasound and/or CT scan of the abdomen were mesenteric lymphadenopathy (5 patients), ascites (5), colitis (3), intestinal loop thickening (3), free fluid in the abdominal cavity (1) and gallbladder thickening (1).

All the patients were hospitalized, with 39 (68%) in a pediatric intensive care unit. The median length of hospital stay was 10 days (range 5–15). A total of 38/57 (67%) of the patients received a combined IVIG and glucocorticoid therapy. Only 12 (21%) patients received IVIG exclusively and 2 (3.5%) patients received glucocorticoid only. Five (9%) patients received none of the above medications (Table 2). When patients who received combination therapy (IVIG plus glucocorticoid) were compared with patients who received only IVIG or glucocorticoid, regarding length of hospital stay and the need for intensive care, there was no statistical difference between the groups relating to length of hospital stay. Conversely, the need for intensive care was more frequent in the patients who received combination therapy than in patients who received only IVIG or glucocorticoid (94.5% vs. 23%, *p* < 0.001). Thirty-one (54%) patients had a shock refractory to the volume replacement and received vasoactive drugs. Seven (12%) patients required mechanical ventilation. In our cohort, no patients received immunobiological agents or cardiopulmonary bypasses. No deaths were observed.

## Discussion

This cohort described the main clinical and laboratory manifestations and treatment used in 57 Brazilian children and adolescents with PIMS-TS who were hospitalized and followed up in five tertiary pediatric rheumatology centers. The most frequent manifestations were fever, gastrointestinal and mucocutaneous. Almost 80% of the patients showed a KD phenotype, either the classic or the incomplete form. All of them presented elevated inflammatory activity markers and most presented altered blood counts, especially anemia and lymphopenia. A laboratory detection of SARS-CoV-2 was found in most patients and after treatment with IVIG and/or glucocorticoids, the patients had an excellent prognosis, with no deaths.

In this study, we used the case definition criteria of the RCPCH as, by their criteria, a laboratory evidence of SARS-CoV-2 and previous contacts with COVID-19 are not mandatory and since not all of our patients had access to laboratory tests for the diagnosis of SARS-CoV-2 [9].

In our cohort, PIMS-TS was observed in all age groups, predominantly between 6–12 years. This finding was also observed in American cohorts and in a systematic review article [19–21]. These data confirm one of the main epidemiological differences between PIMS-TS and KD, which is the age range affected [13, 14]. In KD, the vast majority of children are under 5 years of age, with a median of 3 years [16]. Several publications have described a slight predominance of males over females, which was not observed in our sample. In KD, the greater involvement of males is already well established [16].

The two main comorbidities described in the main multisystem inflammatory syndrome registries are obesity and asthma, which were also described in this study, although less frequently [19–22]. Mortality from acute COVID-19 infection is rare in pediatrics and is associated with the presence of comorbidities, which in PIMS-TS rarely occurs, as it usually affects previously healthy children.

The presence of fever was described in all patients in our cohort, as well as a high frequency of mucocutaneous manifestations, mainly rash, conjunctivitis and hyperemia of the oral mucosa. These are classic findings in KD, and are the main clinical similarities between PIMS-TS and KD [13, 14]. However, as described in other case series from different countries, we also found in our cohort the presence of abdominal manifestations, mainly abdominal pain [4–8]. In some series, some of these patients even underwent an exploratory laparotomy to clarify the abdominal pain [6, 8]. While fever and mucocutaneous manifestations were the main similarities observed between PIMS-TS and KD, the presence of abdominal pain was the main difference between the two diseases, since abdominal pain is rare in KD and is shown to be a frequent manifestation in PIMS-TS [13, 14].

Although both diseases have cardiovascular manifestations, the type and severity of involvement are different between PIMS-TS and KD [13, 14]. As observed in other series, we also observed in our PIMS-TS patients a higher frequency of myocarditis and a lower frequency of coronary artery changes, which are the main feature of KD [8, 19, 20]. Myocardial involvement associated with shock in KD, known as KD shock syndrome, is rare, affecting less than 10% of patients with KD [17]. In other words, in PIMS-TS, a myocardial involvement is common and severe, whereas a coronary artery involvement is mild and transient [23–25].

At the beginning of the pandemic, several case reports and series compared PIMS-TS and KD because of the similarities between the two entities [4, 6, 7]. In an initial series of 10 patients in the province of Bergamo, half of the patients met the criteria for the classic form of KD and the other half for the incomplete form [5]. As the pandemic progressed, the frequency of complete KD was described as more frequent, as occurred in our sample [7, 26]. Although most patients met the classification criteria for KD, PIMS-TS has a wide phenotypic range beyond KD: ranging from multiple organ dysfunction with shock and myocarditis to a febrile phenotype, with more than one mild systemic involvement (especially mucocutaneous or gastrointestinal) and elevation of inflammatory markers [6].

All the patients presented elevated inflammatory markers, especially CRP. In addition, most presented increase in ferritin, LDH, and aminotransferases, which supports PIMS-TS as a hyperinflammatory syndrome. Interestingly, although D-dimer was increased in 96% of the patients in our cohort, no thrombotic events were observed. The frequency of thrombotic events ranged from 0 to 7% in some series [20, 27]. In some inflammatory conditions the level of D-dimer was elevated without the occurrence of thrombotic events [28].

The main changes found in the blood count were anemia and lymphopenia. While anemia reflects an inflammatory activity of PIMS-TS, lymphopenia is the main hematologic feature of the SARS-CoV-2 infection [29]. Unlike the classic finding of thrombocytosis found in KD, only 12% of the patients in our cohort showed an elevation of platelets [16]. However, we observed thrombocytopenia in 30% of cases. This dissociation between the frequency of thrombocytosis classically found in KD and the higher frequency of thrombocytopenia in PIMS-TS has also been observed in other case series and also corroborates that KD and PIMS-TS are two distinct clinical entities [5, 6].

We observed in our cohort that three-quarters of the patients showed evidence of infection with SARS-CoV-2, either by RT-PCR or serology. In a recent systematic review, this frequency was 84.7% [22]. As about one fifth of the patients did not undergo a serology, the lowest frequency of evidence of a previous infection by SARS-CoV-2 found may be justified, since serology has a higher positivity rate than RT-PCR in PIMS-TS [30].

Our pediatric intensive care unit admission rate of 68% was slightly lower than that reported in the literature (71%), however, our patients stayed more days in the hospital (10 days) when compared to the literature (7.9 days) [22]. At the beginning of the case series descriptions, most of the patients were only using IVIG due to the overlap of clinical manifestations between KD and PIMS-TS and consequently the extrapolation of IVIG use in KD [4–6]. Recently, some guidelines and observational studies have advocated, especially in severe cases, the association of IVIG and glucocorticoids [31–35]. In our cohort, we observed a higher frequency in the combined prescription of IVIG and glucocorticoids. These patients also presented a higher frequency of admission to the pediatric intensive care unit but a similar length of hospital stay when compared to patients who received only IVIG or glucocorticoid. These results were expected since the former group presented a more severe disease with need for intensive care. At the same time, they presented a similar outcome to the other group, as there was no difference in the days of hospitalization.

A mechanism of action of IVIG in these patients was recently described. Peripheral blood samples were collected before and after IVIG infusion. It was observed that patients had a high concentration of IL-1β producing neutrophils before IVIG infusion and after infusion there was a marked reduction of these neutrophils, indicating an important anti-inflammatory effect of IVIG by reducing IL-1β production [36, 37]. Furthermore, an in vitro assay demonstrated that IVIG causes the cell death of these neutrophils [36, 37].

Five patients in our cohort received neither IVIG nor glucocorticoid. These patients had a mild clinical phenotype, with only mucocutaneous and gastrointestinal manifestations. None of them required admission to a pediatric intensive care unit. Patients with mild symptoms do not always require immunomodulatory treatment, only close monitoring [31].

This study corroborates the findings in literature on PIMS-TS to date, through a representative sample of Brazilian patients who were followed by pediatric rheumatologists, with extensive experience in inflammatory diseases. However, it presents some limitations due to the retrospective nature of the data collection, such as the non-standardization in the management and the request of complementary exams; no documentation of a history of exposure to SARS-CoV-2, besides the fact that some patients did not perform one of the laboratory tests to confirm SARS-CoV-2 infection. For this reason, it was not possible to use the CDC [10] or the WHO [11] criteria, and the RCPCH criteria were used for PIMS-TS case definition [9].

## Conclusions

This study evidenced the high morbidity of PIMS-TS in Brazilian children, with a high rate of pediatric intensive care unit admission and a prolonged length of stay, which can generate a significant economic and social impact. Larger multicenter and follow-up studies of these patients are needed to assess the morbidity of the disease in the medium and long term.

## Data Availability

All data generated during this study are included in this article or are available from the corresponding author on reasonable request.

## Abbreviations

SARS-CoV-2: Severe acute respiratory syndrome coronavirus 2
PIMS-TS: Paediatric inflammatory multisystem syndrome temporally associated with SARS-CoV-2
RCPCH: Royal College of Paediatrics and Child Health
CRP: C-reactive protein
IVIG: Intravenous immunoglobulin
COVID-19: Coronavirus disease 2019
KD: Kawasaki disease
ESR: Erythrocyte sedimentation rate
LDH: Lactate dehydrogenase
SD: Standard deviation
IQR: Interquartile range
